# Hospital Admissions Associated With Noncommunicable Diseases During the COVID-19 Outbreak in Brazil

**DOI:** 10.1001/jamanetworkopen.2021.0799

**Published:** 2021-03-08

**Authors:** Jean Henri Maselli-Schoueri, Luis Eduardo Werneck de Carvalho, Leandro F. M. Rezende, Fernando Luiz Affonso Fonseca, Gerson Ferrari, Fernando Adami

**Affiliations:** 1 Laboratório de Epidemiologia e Análise de Dados, Centro Universitário Saúde ABC, Santo André, São Paulo, Brasil; 2 Oncológica do Brasil Ensino e Pesquisa, Belém, Pará, Brasil; 3 Universidade Federal de São Paulo, Escola Paulista de Medicina, Departamento de Medicina Preventiva, São Paulo, Brasil; 4 Laboratório de Análises Clínicas, Centro Universitário Saúde ABC, Santo André, São Paulo, Brasil; 5 Universidad de Santiago de Chile, Escuela de Ciencias de la Actividad Fisica, el Deporte y la Salud, Chile

## Abstract

This cross-sectional study uses data from the Brazilian Unified Health System to compare the number of hospital admissions for treatment of noncommunicable diseases from January to June 2020 with that during the corresponding period in each of the previous 3 years in São Paulo, Brazil.

## Introduction

High-income countries have reported reductions in hospital admissions associated with cardiovascular diseases (CVDs) during the coronavirus disease 2019 (COVID-19) pandemic.^1,2^ However, the overall association of the COVID-19 pandemic with hospital admissions for noncommunicable diseases (NCDs) in low- and middle-income countries is still unclear. We assessed the number of hospital admissions for neoplasms, metabolic diseases, CVDs, and musculoskeletal diseases in São Paulo, Brazil, between January and June 2020 compared with the corresponding periods in the previous 3 years.

## Methods

In this cross-sectional study, hospital admissions for NCDs were obtained from the Hospital Information System, a publicly available official database of hospital admissions in the Brazilian Unified Health System (Sistema Único de Saúde). The number of hospital admissions for neoplasms (C00-D48), metabolic diseases (E00-E90), CVDs (I00-I99), and musculoskeletal diseases (M00-M99), according to the *International Statistical Classification of Diseases and Related Health Problems, Tenth Revision*, was collected from January to June of each year from 2017 to 2020. Data were collected by 2 of the authors, and any incompatibility led to a new collection of data. Because the study used secondary data, ethical approval and informed consent were not required according to resolution 510 of the Brazilian National Health Council. This study followed the Strengthening the Reporting of Observational Studies in Epidemiology (STROBE) reporting guideline.

Linear regression was used to describe the changes in hospital admissions throughout the selected months for each year. We also compared the number of hospital admissions in June 2020 vs January 2020 (before the first case of COVID-19 was reported in São Paulo on February 26, 2020). Statistical significance was set at 2-tailed *P* < .05, and data analysis was performed using Stata, version 11.0 (StataCorp LLC).

## Results

The number of hospital admissions for NCDs between January and June was stable from 2017 to 2019. However, we observed a decrease in the absolute numbers of hospital admissions for NCDs between January and June 2020, with mean reductions in hospital admissions per month of 505 (95% CI, 126-884) for CVDs, 332 (95% CI, 95-569) for neoplasms, 136 (95% CI, 46-227) for musculoskeletal diseases, and 76 (95% CI, 1-151) for metabolic diseases (Table). During June 2020 compared with January 2020, there was a decrease of 543 hospital admissions (68%) for musculoskeletal diseases, 332 admissions (44%) for metabolic diseases, 2129 admissions (38%) for CVDs, and 1454 admissions (35%) for neoplasms (Figure).

**Table.** Linear Regression of Hospital Admissions in São Paulo, Brazil, From January to June of Each Year, 2017-2020, by Noncommunicable Disease Type

Disease type	β (95% CI)^a^	Hospital admissions, No.	*P* value
Neoplasms			
2017	−2 (−230 to 227)	22 246	.98
2018	37 (−89 to 162)	21 527	.46
2019	−21 (−165 to 123)	23 068	.71
2020	−332 (−569 to –95)	19 192	.02
Endocrine, nutritional, and metabolic diseases			
2017	3 (−30 to 36)	4006	.82
2018	−10 (−29 to 9)	4145	.23
2019	−8 (−35 to 20)	4385	.47
2020	−76 (−151 to −1)	3060	.049
Diseases of the circulatory system			
2017	139 (−140 to 417)	33 632	.24
2018	48 (−128 to 223)	33 264	.49
2019	−42 (−212 to 129)	32 001	.54
2020	−505 (−884 to −126)	24 524	.02
Diseases of the musculoskeletal system and connective tissue			
2017	17 (−27 to 60)	4752	.34
2018	−1 (−34 to 32)	4598	.95
2019	−1 (−24 to 22)	4640	.92
2020	−136 (−227 to −46)	2780	.01

^a^ Estimated change in the mean number of hospital admissions between January and June of each year.

**Figure.**
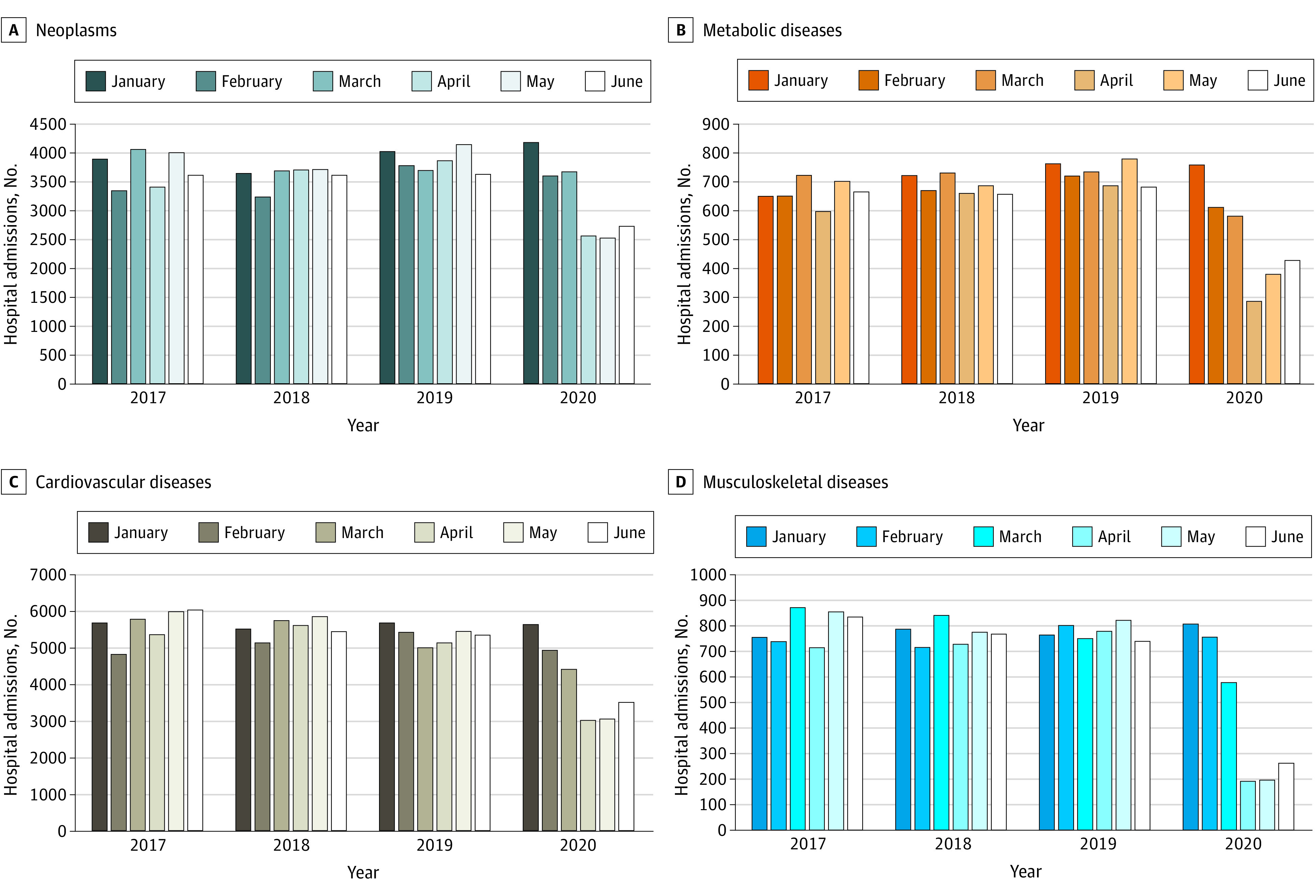
Hospital Admissions by Noncommunicable Disease Type From January to June of Each Year, 2017-2020, São Paulo, Brazil Disease types are based on *the International Statistical Classification of Diseases and Related Health Problems, Tenth Revision*.

## Discussion

In this cross-sectional study, we observed a reduction in hospital admissions for NCDs from January to June 2020 compared with the corresponding period in each of the 3 previous years in São Paulo, Brazil. Social and physical distancing measures and the fear of severe acute respiratory syndrome coronavirus 2 (SARS-CoV-2) infection may have been associated with the reduction. A decrease in hospital admissions could be associated with an increase in the number of deaths and complications owing to a lack of medical follow-up. Future studies on the association between the COVID-19 pandemic and deaths from NCDs are warranted.

This study has limitations, including its cross-sectional design and the possibility of incomplete or inaccurate registration of hospital admissions in the official hospital admission system during the pandemic. To minimize these issues, we compared data from the first 6 months of 2020 with those from the same period in each of the previous 3 years. To our knowledge, few studies have quantified the change in hospital admissions for NCDs during the COVID-19 pandemic in low- to middle-income countries.^3,4^ In the US, however, studies^5^ have reported that patients not infected with SARS-CoV-2 may have avoided medical care because of fear or the recommendation of a health care professional, both of which should be assessed to avoid future complications.^6^ This study’s results may be considered by decision-makers when planning the hospital capacity of the Brazilian Unified Health System for treatment of NCDs. The reduction in hospital admissions observed during the pandemic could lead to a collapse of the hospital system because of an increase of patients with worse clinical conditions. Adequate funding of the Brazilian Unified Health System is imperative to address the COVID-19 pandemic and the potential indirect association of the pandemic with the number of admissions for NCDs.
